# Phylodynamic analysis of porcine circovirus type 2: Methodological approach and datasets

**DOI:** 10.1016/j.dib.2016.06.005

**Published:** 2016-06-15

**Authors:** Giovanni Franzo, Martì Cortey, Joaquim Segalés, Joseph Hughes, Michele Drigo

**Affiliations:** a Department of Animal Medicine, Production and Health (MAPS), University of Padua, Viale dell’Università 16, 35020 Legnaro (PD), Italy; b Centre de Recerca en Sanitat Animal (CReSA), UAB-IRTA, Barcelona, Spain; c MRC-University of Glasgow Centre for Virus Research, Glasgow, Scotland, UK

## Abstract

Since its first description, PCV2 has emerged as one of the most economically relevant diseases for the swine industry. Despite the introduction of vaccines effective in controlling clinical syndromes, PCV2 spread was not prevented and some potential evidences of vaccine immuno escape have recently been reported (“Complete genome sequence of a novel porcine circovirus type 2b variant present in cases of vaccine failures in the United States” (Xiao and Halbur, 2012) [1], “Genetic and antigenic characterization of a newly emerging porcine circovirus type 2b mutant first isolated in cases of vaccine failure in Korea” (Seo et al., 2014) [2]). In this article, we used a collection of PCV2 full genomes, provided in the present manuscript, and several phylogentic, phylodynamic and bioinformatic methods to investigate different aspects of PCV2 epidemiology, history and evolution (more thoroughly described in “**PHYLODYNAMIC ANALYSIS of PORCINE CIRCOVIRUS TYPE 2 REVEALS GLOBAL WAVES of EMERGING GENOTYPES and the CIRCULATION of RECOMBINANT FORMS”**[3])**.** The methodological approaches used to consistently detect recombiantion events and estimate population dymanics and spreading patterns of rapidly evolving ssDNA viruses are herein reported. Programs used are described and original scripts have been provided. Ensembled databases used are also made available. These consist of a broad collection of complete genome sequences (i.e. 843 sequences; 63 complete genomes of PCV2a, 310 of PCV2b, 4 of PCV2c, 217 of PCV2d, 64 of CRF01, 140 of CRF02 and 45 of CRF03.), divided in differnt ORF (i.e. ORF1, ORF2 and intergenic regions), of PCV2 genotypes and major Circulating Recombinat Forms (CRF) properly annotated with respective collection data and country. Globally, all of these data can be used as a starting point for further studies and for classification purpose.

**Specifications** **Table**

**Table:** 

Subject area	Biology, Genetics and Genomics
More specific subject area	Phylogenetics and Phylogenomics
Type of data	Excel file
How data was acquired	Sequence data were downloaded from GenBank, manually checked and annotated. Analysis were performed using state of art freely available programs for phylogeny, population dynamics and selective pressure analysis.
Data format	Raw, filtered
Experimental factors	PCV2 complete genome sequences were downloaded from Genbank and annotated with the respective collection country and data. Sequences have been aligned and the consistency of the alignment was checked. All sequences were scanned for recombination and subdivided in genotypes or recombinant forms. Databases generated in this way were used for further analysis.
Experimental features	PCV2 sequences download, quality check and annotation.
	Sequence alignment, recombination analysis, Coalescent based analysis of population parameters and reconstruction of viral spreading patterns. Analysis of selective pressure acting on different coding regions.
Data source location	n/a
Data accessibility	Data are within the article

**Value of the data**

• Most extensive collection of PCV2 full genome sequences with available metadata.
• Proper annotation linking genetic data to country of origin and collection data
• Full description of several approaches used to analyze different aspects of viral evolution
• Datasets suitable for further evolutionary studies and for PCV2 classification purpose
• Standardized approach that can be used for follow-up studies on PCV2 evolution.

## 1 Data

Supplementary data 1 provides a table reporting the accession number of all (i.e. 843) PCV2 complete genomes and PCV2a ORF2 sequences used in Franzo et al. [3]. For each sequence, the country where it has been sampled and the collection data are also reported. The alignments of all major PCV2 genotypes (i.e. PCV2a, PCV2b, PCV2c and PCV2d) and circulating recombinant forms (CRF) are provided in Supplementary data 2 and could be used for comparison purpose and as a starting point for further studies. Finally, Supplementary data 3 provides an R script for ancestral state reconstruction of per-site amino acid sequence using a maximum likelihood approach.

## 2 Experimental design, materials and methods

### 2.1 Dataset

A total of 925 PCV2 complete genome sequences with known collection dates and country of origin were downloaded from GenBank (accessed 06/10/2014 – listed in “Supplementary data 1.xls” in the online version of this article) and aligned using the MAFFT method [4]. All poorly aligned sequences and those displaying degenerate nucleotides or indels which caused reading frame alterations, suggesting sequencing errors, were removed from the dataset (898 sequences were maintained) (Supplementary data 1).

#### 2.1.1 Recombination analysis

The whole dataset was tested for recombination using two programs based on different approaches: RDP4 [5] and GARD [6]. When RDP was used, only recombination events detected by more than 2 methods with a significance value lower than 10^−5^ (*p*-value <10^−5^) and Bonferroni correction were accepted. The non-recombinant sequences as well as those sharing recombination events were split into separate datasets and expanded to their original size.

### 2.2 Genotyping and database preparation

The non-recombinant sequences were classified into genotypes PCV2a, PCV2b, PCV2c or PCV2d according to Franzo et al. 2015 [7].

The most appropriate nucleotide substitution model was selected according to the results of the Akaike information criterion (AIC) score calculated using JModel Test 2.1.2 [8]. A phylogenetic tree was reconstructed using the Maximum likelihood (ML) approach implemented in PhyML [9]. The best tree search method included the combination of two branch swapping algorithms: nearest neighbor interchange (NNI) and subtree pruning and regrafting (SPR). The robustness of the monophyly of the taxa subsets was estimated with the fast non-parametric version of the aLRT (Shimodaira–Hasegawa [SH]-aLRT), developed and implemented in PhyML 3.0 [10]. On the basis of the recombination and phylogenetic analyses, sequences were divided into independent datasets, corresponding to different genotypes and CRFs (i.e. those including more than 30 sequences collected in two or more countries). Every dataset was further divided in three regions, namely *ORF1*, *ORF2* and intergenic region (obtained merging together the major and the minor intergenic regions) and a new alignment was generated on each dataset. The coding regions were aligned at the amino acid level and then the nucleotide sequences were back-translated using the MAFFT algorithm implemented in TranslatorX [11]. All these datasets, comprising different gene alignments, are provided in Supplementary data 2. These include 63 complete genomes of PCV2a, 310 of PCV2b, 4 of PCV2c, 217 of PCV2d, 64 of CRF01, 140 of CRF02 and 45 of CRF03. Additionally a dataset of 83 PCV2a ORF2 sequences is provided.

### 2.3 BEAST and selective pressures analysis

The time to most recent common ancestor (tMRCA), substitution rates, phylogeography and population dynamics were jointly estimated using a Bayesian serial coalescent approach implemented in BEAST 1.8.1 [12].

The selective pressure on the viral proteins was estimated using different methods based on the ratio between non-synonymous and synonymous substitution rates (dN/dS).

Pervasive diversifying/purifying selection was estimated using SLAC, FEL and FUBAR method while episodic diversifying selection was evaluated using MEME [13], [14], [15]. The action of selective pressures was compared among different genes using the *dNdSDistributionComparison.bf* implemented in HyPhy [16]. Differences in the site-by-site selection patterns among different genotypes were investigated for each gene using the batch files *CompareSelectivePressure.bf* implemented in the same program. Ancestral state reconstruction of per site amino acid sequence was performed, based on the time scaled phylogenetic trees, using the maximum likelihood approach of the *ape* package implemented in R [17].The corresponding script is provided in Supplementary data 3.

## Footnotes

Transparency document Transparency data associated with this article can be found in the online version at doi:10.1016/j.dib.2016.06.005.
